# Mechanical Behavior of Alkasite Posterior Restorations in Comparison to Polymeric Materials: A 3D-FEA Study

**DOI:** 10.3390/polym14081502

**Published:** 2022-04-07

**Authors:** Pietro Ausiello, Amanda Maria de Oliveira Dal Piva, Alessandro Espedito di Lauro, Franklin Garcia-Godoy, Luca Testarelli, João Paulo Mendes Tribst

**Affiliations:** 1 School of Dentistry, University of Naples Federico II, Via S. Pansini 5, 80131 Naples, Italy; alessandroespedito.dilauro@unina.it; 2 Academic Centre for Dentistry Amsterdam (ACTA), Department of Dental Materials, University of Amsterdam and Vrije Universiteit Amsterdam, 1081 LA Amsterdam, The Netherlands; amodalpiva@gmail.com (A.M.d.O.D.P.); joao.tribst@gmail.com (J.P.M.T.); 3 Department of Bioscience Research, College of Dentistry—University of Tennessee Health Science Center, Memphis, TN 38163, USA; fgarciagodoy@gmail.com; 4 Department of Oral and Maxillofacial Sciences, La Sapienza University of Rome, 00161 Rome, Italy; luca.testarelli@uniroma1.it

**Keywords:** dental restoration failure, resin composite, finite element analysis, dental materials

## Abstract

The present investigation evaluated the effect of the combination of different dental filling materials in Class I cavities under occlusal loading using three-dimensional finite elements analysis (FEA). Six computer-generated and restored models of a lower molar were created in the CAD software and compared according to the biomechanical response during chewing load condition. Two adhesively bonded bulk restorative materials [bulk-fill resin composite (BF) or Alkasite (Alk)] were evaluated with or without the presence of a base material below (flowable resin composite or glass ionomer cement). A food bolus was placed on the occlusal surface mimicking the compressive occlusal load (600 N) during the static linear analysis. The maximum principal stress (tensile) was calculated as stress criteria in enamel, dentin and restoration. All models showed high stresses along the enamel/restoration margin with a similar stress trend for models restored with the same upper-layer material. Stress values up to 12.04 MPa (Alk) or up to 11.12 MPa (BF) were recorded at the enamel margins. The use of flexible polymeric or ionic base material in combination with bulk-fill resin composite or Alk did not reduce the stress magnitude in dentine and enamel. Class I cavities adhesively restored with bulk-fill resin composite showed lighter stress concentration as well as Alk. Therefore, adhesively bonded Alk restoration showed a promising mechanical behavior when used with different base materials or as a bulk restoration for posterior Class I cavity.

## 1. Introduction

Composite resin-based dental fillings are assumed to be advanced polymeric materials in adhesive dentistry due to their optimal properties that mechanically and aesthetically replace missing dental tissues [1,2]. These materials are used in daily dentistry as direct fillings when an effective restoration of weakened or fractured teeth is required [3]. Despite that, the organic matrix, residual monomers and the potential cytotoxic risk of the resin composites components are still investigated in the literature [4,5].

A greater clinical application of new and modified resin monomers as low stressing bulk dental materials is advocated to restore deep posterior cavities [6]. Leakage effects associated with the stressing and shrinking of resin composites in adhesively bonded posterior restorations are consequently reduced [7]. In this way, the residual polymerization shrinkage stress following the photo-polymerization kinetic is more deeply linked to the polymer reaction characteristics and the C-factor influence can be severely reduced [8]. In addition, upon investigating the stress distribution in large posterior restorations, a more favorable behavior has been observed when glass ionomer cement is layered in combination with a resin filling composite in comparison to a bulk-fill resin composite filling [9].

Another alternative to restore large cavities in molars has been advocated to be the use of a modified resin composite with alkaline filling [10]. Cention N (Ivoclar, Lichtenstein) has been developed with the main purpose to replace amalgam material. It is a bulk-fill material available for both dual- and self-cure modes with promising chemical and physical properties as a direct dental material. Previous results for microleakage [11,12], flexural strength [13,14], shear bond strength [14,15], compressive strength [12,14], and microhardness [12,13,14,15] indicate that direct filling restorative material is promising. Based on its mechanical [12,13] and optical properties added to its bioactive properties of ion release (calcium, hydroxyl, and fluoride), it is indicated to stabilize oral pH and form apatite [16] by reducing demineralization and inducing dental remineralization. Cention N has also been directly compared to glass ionomer cements [13,14,16]. Differently from these materials, it can be indicated for long-term restorations due to its wear resistance. Based on its indications and due to its use as bulk material for large cavities, it has been compared to bulk-fill dental filling polymeric materials.

Cention N is an Alkasite [Alk] in a new category of filling material with setting reaction of four minutes, starting after the mixing of powder and liquid. Cention N contains Ivocerin as a photoinitiator and an acyl phosphine oxide initiator [14], as well as calcium fluorosilicate glass [16]. Therefore, this restorative material is self-curing with optional additional light-curing, indicated for the basic filling combining bulk placement, ion release, durability, and esthetics [13]. After curing, the pH value during acid attacks is regulated by increased hydroxide ion release from the alkaline filler [12]. Cention N satisfies the minimum ISO 4049 value without difference for self-cured and light-cured modes, thus making it a promising material in stress-bearing areas [15].

However, the mechanical behavior of bulk-fill restorations in resin composites or in Alk for molar cavities has not been investigated yet. Thus, is there any mechanical benefit that can result in the material clinical choice for Class I cavities in molars? In addition, what is the mechanical effect in the restoration when different base materials are used in combination with Alk?

Finite element analysis has been widely used to investigate bulk-fill restorations behavior [9] and more recently Alk performance for Class V cavities [9,17,18]. Therefore, by means of 3D FEA, this study aims to investigate the mechanical behavior of different bulk-fill materials with and without the presence of a polymeric or a ionic base material in molar Class I restorations. The null hypothesis was that the different filling restorative materials would not influence the restoration mechanical behavior.

## 2. Materials and Methods

**Figure 1:**
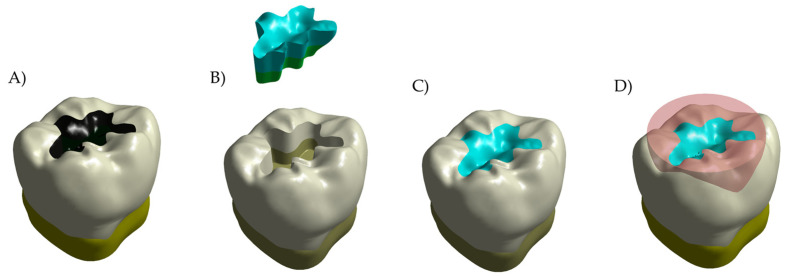
Schematic illustration of the simulated conditions. (**A**) Decayed tooth model, (**B**) model of a restoration (**above**) and tooth with a Class I cavity (**below**), (**C**) restored tooth, and (**D**) restored tooth with the food bolus at the occlusal surface as loading condition.

The present study applied the computer-aided design and finite element method (CAD-FEM) as a bioengineering tool to calculate the stress distribution in molar Class I restorations. This method has been extensively applied to investigate the mechanical behavior in different dental fields [2,6,9,17,18,19,20], including operative dentistry [2], dental materials evaluation [6], restoration concepts in molars [9,19] and premolars [17], and implant therapy [20]. The three-dimensional (3D) model definition is presented in Figure 1.

**Figure 2:**
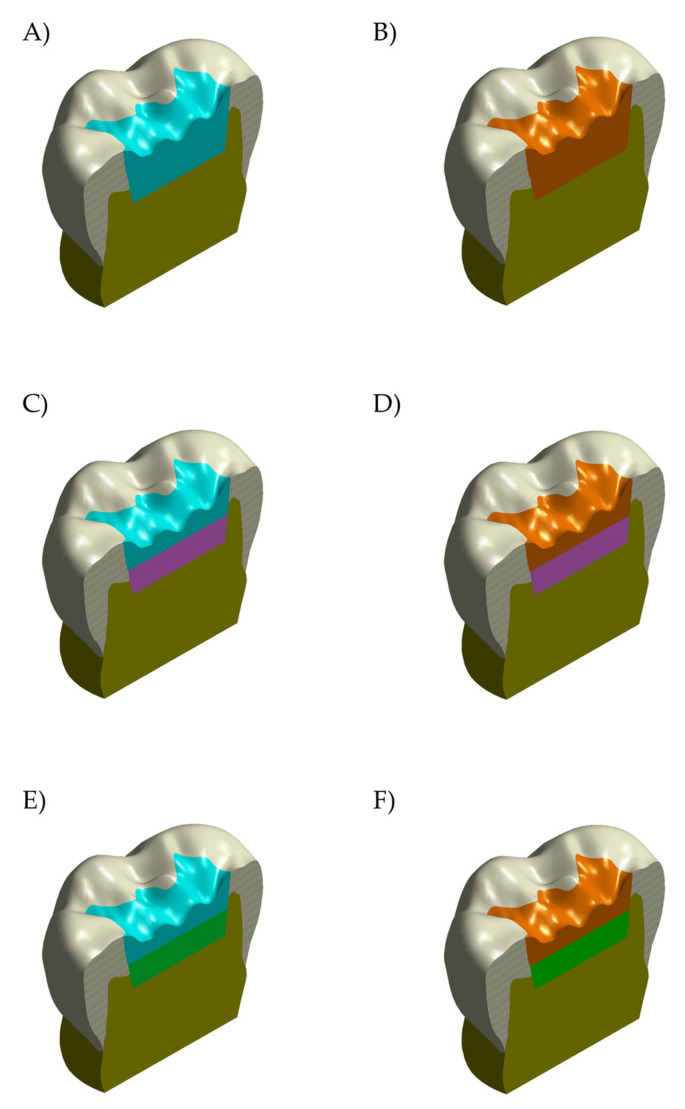
The geometric features of the analyzed model-cavity according to the filling: bulk restoration in (**A**) bulk-fill resin composite or (**B**) Alkasite; flowable resin composite below (**C**) bulk-fill resin composite or (**D**) alkasite; glass ionomer cement below (**E**) bulk-fill resin composite or (**F**) Alkasite.

The previously created 3D CAD model of a sound molar [2,9,21] was considered to design a model with Class I cavity (Figure 1A). The lower molar was digitalized with a high resolution micro-CT scanner system (Bruker microCT), and dentin and enamel tissue volumes were obtained. The data sets were processed with InVesalius 3.1.1 software and 3D tessellated surfaces were generated with cross-section curves. Then, the parametric 3D model was created using loft surfaces, and the subtractive Boolean was used to ensure the juxtaposition of contacting surfaces between dentin and enamel. In sequence, the obtained tooth model was sectioned 2.5 mm below the cervical area and placed in a special coordinate system (*X*-axis and *Y*-axis were used for the bucco-lingual direction, while the *Z*-axis was oriented upwards) [2]. The final dimensions of the tooth were 10.60 mm bucco-lingually and 12.36 mm mesio-distally. The model was prepared with a Class I cavity which presents the cavity floor and axial walls with rounded angles. Finally, the model was replicated in 6 different conditions according to the restorative materials [21]. The mechanical responses of the adhesively bonded materials as bulk restoration [bulk-fill resin composite (BF) or Alkasite (Alk)] or combined with other base materials (flowable resin composite or glass ionomer cement) were evaluated. Therefore, six different models of restoration were considered, as presented in Figure 2.

**Figure 3:**
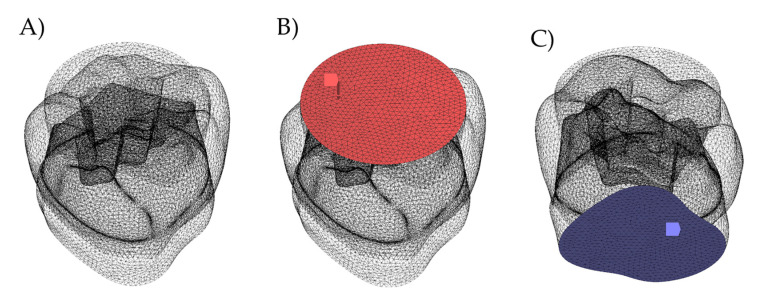
Boundary conditions applied in the present simulation. (**A**) meshing model, (**B**) compressive axial (red arrow) loading applied through the simulated food bolus (red) on the occlusal surface, and (**C**) fixation support (purple surface).

To simulate the masticatory loads variability that is affected by the contact between tooth and food bolus, a solid volumetric model of food on the occlusal surface was also designed (Figure 1D). This loading approach has been previously applied in studies that evaluated similar conditions [2,9,19,20,21,22]. The restored models description was summarized in Table 1, and the geometrical features of the restoration conditions are shown in Figure 3.

The response of the six restored models was assessed by the computer-aided engineering software (ANSYS 19.2, ANSYS Inc., Houston, TX, USA). All models were discretized using 4-node tetrahedral elements with a total size extending from 0.08 mm to 0.18 mm. To minimalize the mesh effect in the stress results, caused by the small curvature radius and notch effects, mesh improvement techniques were used with a mesh convergence test considering von-Mises stress maximum values. The analyses were based on the loading during the maximum bite force at the chewing cycle [8,9]. The food bolus was positioned on the occlusal surface and a slide-type contact was used. The total number of elements and nodes for the bulk model was 445,242 with 97,364 nodes, respectively, while the model with two different restorative materials presented 438,092 elements with 97,412 nodes.

**Figure 4:**
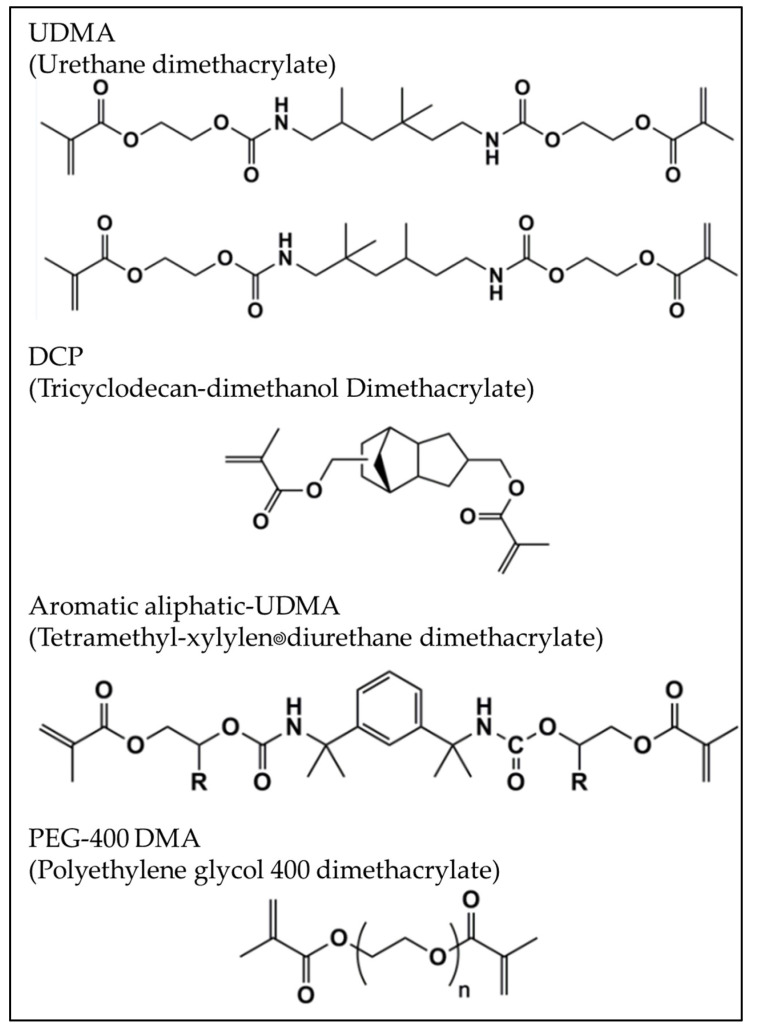
Structural formulae of monomers present in the evaluated Alk. Adapted from the manufacturer’s scientific documentation (Cention N, Ivoclar, Lichtenstein) [23].

The present simulation did not consider the polymerization shrinkage stresses in the resin based materials due to the lack of data for Alk. Therefore, as a limitation, the kinetic stress relaxation was considered insignificant and only the elastic modulus and Poisson ratio were applied as the constant elastic properties for the stress calculation. The chemical formula for the monomers presented in the experimental material (Alk) are summarized in Figure 4 [23].

The physiological masticatory load (600N) was simulated during the occlusal static load applied to the food bolus. The lower surface of the models was constrained in all directions (Figure 3). Statistical and linear analyses were carried out and performed considering a non-failure condition in the elastic limit of each material.

The calculated stress magnitude for the models were qualitatively and quantitatively compared. Assuming that these materials exhibit brittle mechanical behavior [9], the first principal stress was calculated for enamel, dentine, restoration and cavity margin.

## 3. Results

**Figure 5:**
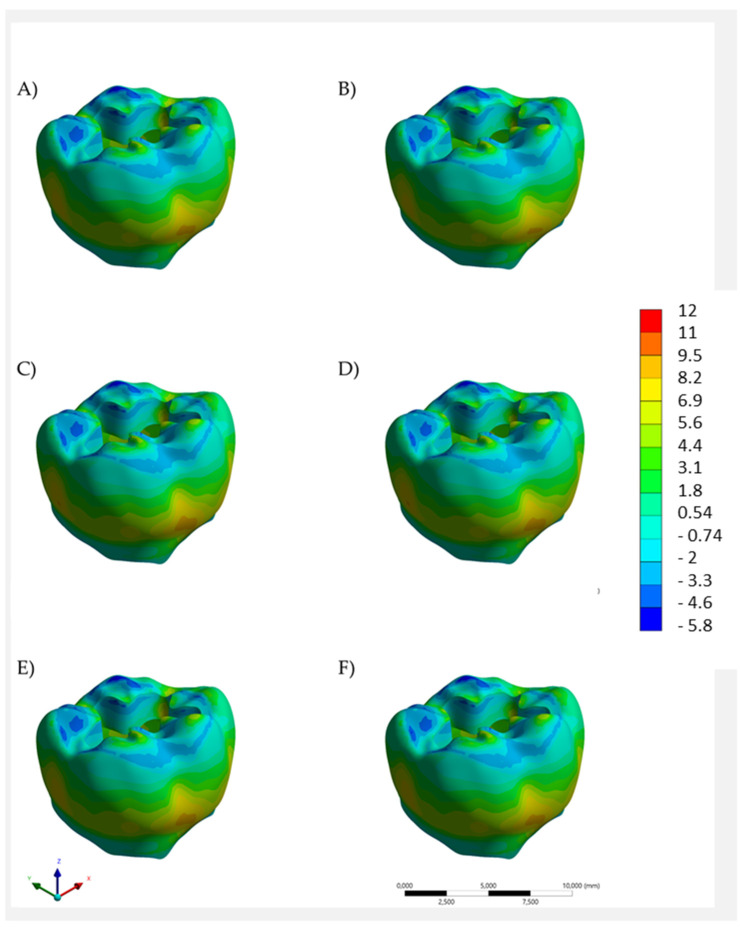
Tensile stress maps in enamel according to the restorative material(s): bulk restoration in (**A**) bulk-fill resin composite or (**B**) Alkasite; flowable resin composite below (**C**) bulk-fill resin composite or (**D**) Alkasite; glass ionomer cement below (**E**) bulk-fill resin composite or (**F**) Alkasite.

**Figure 6:**
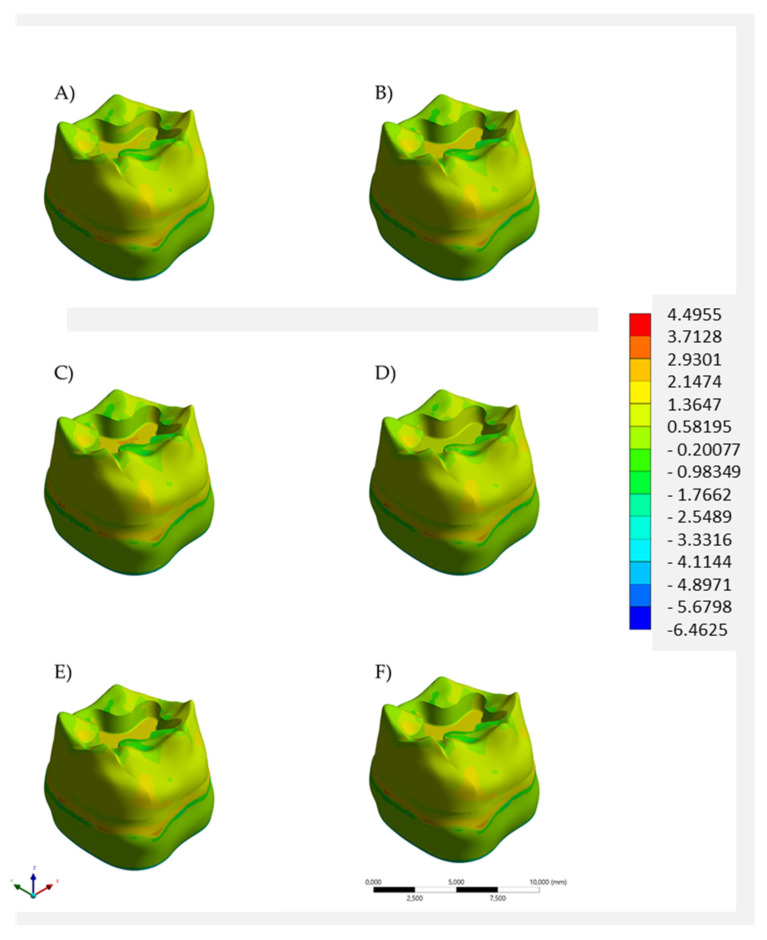
Tensile stress maps in dentin according to the restorative material(s): bulk restoration in (**A**) bulk-fill resin composite or (**B**) Alkasite; flowable resin composite below (**C**) bulk-fill resin composite or (**D**) Alkasite; glass ionomer cement below (**E**) bulk-fill resin composite or (**F**) Alkasite.

Regardless of the restorative material combination, the models exhibited a similar stress trend along the evaluated structures. For both enamel (Figure 5) and dentin (Figure 6), the stress color maps showed similar mechanical behavior for all models. The quantitative analysis of tensile stress peaks (Table 2) showed that the lowest stress magnitude was calculated for the bulk restoration in bulk-fill resin composite (11.12 MPa in enamel and 4.15 MPa in dentine). Bulk restoration in Alk presented a stress peak that was 7.6% higher for both enamel and dentin (12.04 MPa and 4.49 MPa, respectively). Regardless of the base material below, both upper-layer restorative materials were able to promote lower stress peak values.

**Figure 7:**
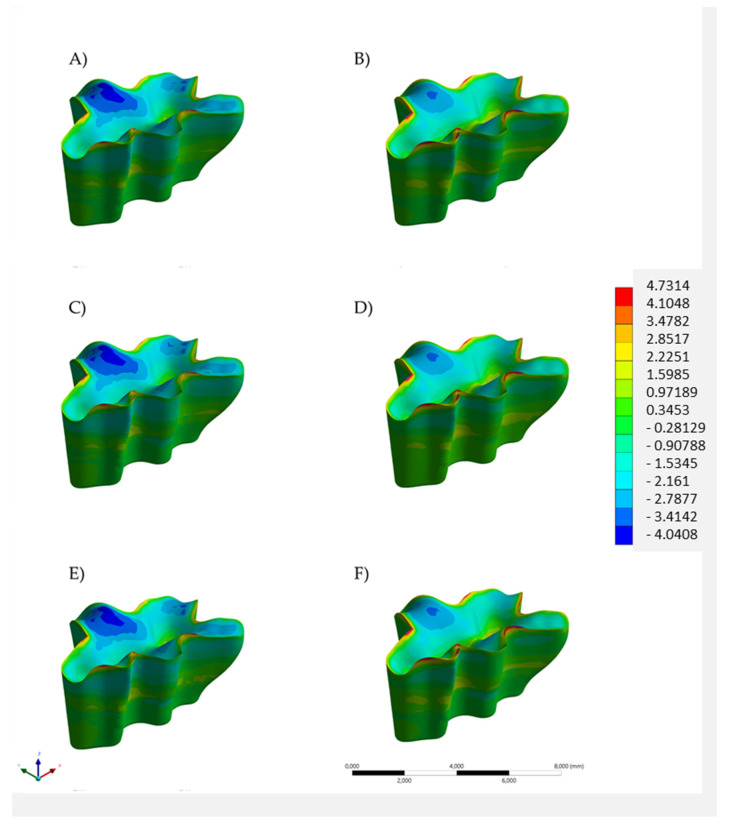
Tensile stress maps in the restoration volume according to the restorative material(s): bulk restoration in (**A**) bulk-fill resin composite or (**B**) Alkasite; flowable resin composite below (**C**) bulk-fill resin composite or (**D**) Alkasite; glass ionomer cement below (**E**) bulk-fill resin composite or (**F**) Alkasite.

**Figure 8:**
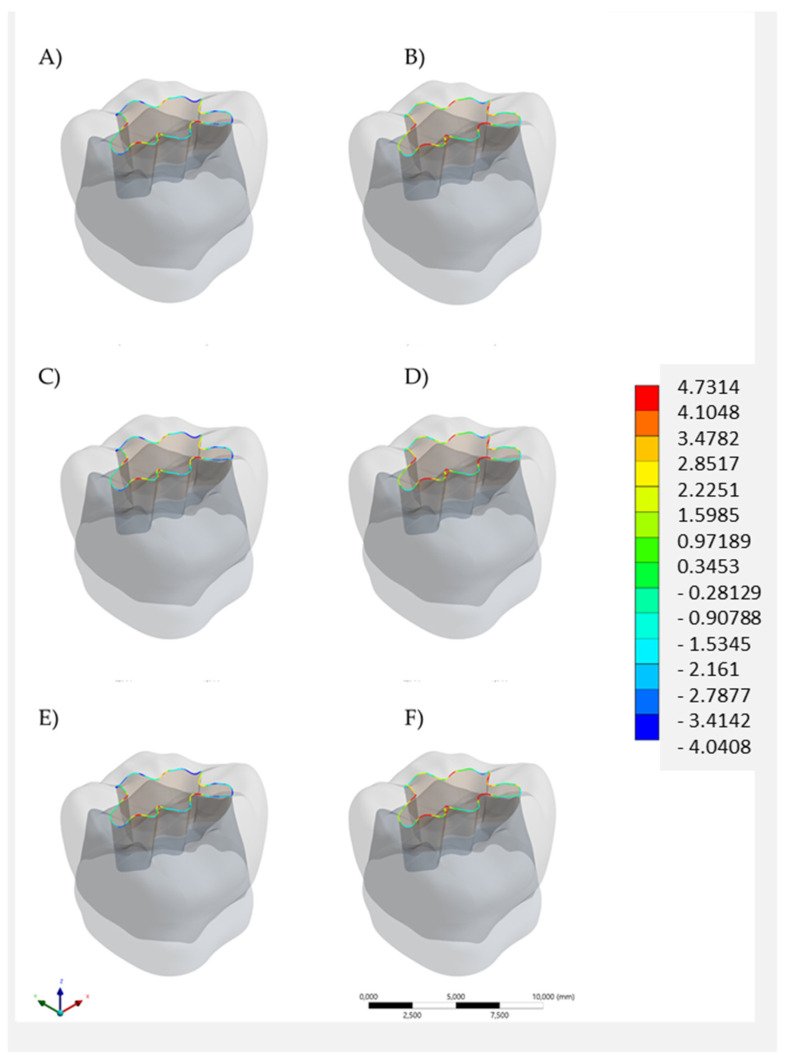
Tensile stress maps in the restoration margin according to the restorative material(s): bulk restoration in (**A**) bulk-fill resin composite or (**B**) Alkasite; flowable resin composite below (**C**) bulk-fill resin composite or (**D**) Alkasite; glass ionomer cement below (**E**) bulk-fill resin composite or (**F**) Alkasite.

For the restoration (Figure 7 and Figure 8), color maps suggest similarity between the models, except in the loading point (Figure 7) and margin (Figure 8), which had better stress distribution for bulk-fill resin composite. Considering the stress peaks (Table 2), an inverse mechanical behavior was noticed. All conditions with the highest stress magnitude in enamel showed lower stress magnitude in the restoration. Thus, the lowest stress peak in the restoration was observed for the bulk restauration in Alk. Moreover, for the restoration, regardless the base material below, the lowest stress peaks were observed when Alk was used.

## 4. Discussion

This study aimed to evaluate the mechanical behavior of different bulk-fill materials with and without the presence of a base material to restore a posterior Class I. Results showed differences between the materials behavior, rejecting the null hypothesis.

When a single-increment restoration was simulated in the present study, similar biomechanical behavior was observed between the BF and the Alk groups, with a numerical difference of 0.92 MPa, not appreciated in Figure 5 and Figure 6 in terms of tensile stress map distribution. Therefore, both polymeric- and ionic-based materials showed an equivalent behavior when used as bulk dental fillings in adhesive conditions. In practice, the mechanical resistance and esthetics (limited for Alk) influence the material selection by the dentist, as well as the possibility of using a bioactive material. According to the manufacturer, Alk filling can be used as self-adhesive bulk material in retentive cavities or in association with an adhesive layer. A previous study has compared the Alk with several bulk materials and it observed the highest shear bond strength value (33.8 MPa) for the Alk in adhesively bonded condition. When Alkasite was applied without adhesive, it critically showed only 3 MPa as a bond strength [15]. Therefore, the present study considered the condition with a proper adhesion between the restoration and cavity walls, and a non-retentive preparation design was simulated. It is important to reinforce that the manufacturer recommends the self-adhesive mode only for retentive cavities, e.g., replacing an old amalgam restoration.

Another study a found similar and higher bond strength, after 14 days, for Alk (14.38 MPa) and nano-hybrid composites compared to glass ionomer cement (5.96 MPa) [14]. In addition, the immediate and aged bond strength to dentin was evaluated for a self-adhesive bulk-fill resin composite, a resin-modified glass ionomer, and an adhesively bonded dual-cure Alk. The authors detected that Alk showed the highest mean values for flat or Class I cavity [24]. However, no data which compare adhesively bonded bulk-fill resin composite and Alk bond strength are available. Results presented in this study suggest that Alk presents a similar stress distribution all along the cavity margins when compared to bulk resin composites. These data suggest that further in vitro bond strength studies with Alk in posterior restorations should be performed to help clinicians choose between both materials.

In terms of flexural strength, self-cured and dual-cured Alk have also been compared to four conventional resin composites and two bulk-fill resin composites. Authors found lower and similar flexural strength for Tetric N-Ceram bulk-fill (103.7 MPa) and dual-cured Alk (96.4 MPa), i.e., higher than self-cured Alk (82.1 MPa). However, the strength significantly decreased after 12 months (71.9 MPa; 53.9 MPa) [25]. Other authors reported no difference in flexural strength according to the cure mode [15,26], even under bleaching regimens [27]. The present study confirms that, in adhesive simulated conditions, there is a similar stress trend between both materials (BF or Alk), regardless of the use of more flexible base material, which did not contribute to better stress relief inside the restoration.

In addition to the colorimetric graphics, a quantitative analysis was carried out in order to compare the restorations by several authors [28,29,30]. Lower tensile stress peaks caused by the loading for enamel and dentin tissues were observed in bulk restorations; however, a low difference (≈7.6%) between the materials peaks was detected. In addition, regardless the presence of a base material, lower stress peaks were observed for both enamel and dentin when the bulk-fill resin composite was used. Considering the small peaks difference between models, results suggest that for both enamel and dentin, the clinicians should also consider other properties in their choice, e.g., the need for ion release in high-caries-risk patients. Many studies have reported the ability for Alk to inhibit caries at restorations margin [31]. In addition, less microleakage was reported for bonded Alk [32]. In this study, it was confirmed that bonded Alk restoration presents an adequate load stress distribution deeply associated with the bonding condition.

Previous investigations with a similar methodology have applied the maximum principal stress as the analysis criteria for the failure of dental materials [2,19,22]. This is indicated based on the failure mode of brittle materials, caused by high tensile stress concentration in regions that are prone to initiate crack propagation or interfacial debonding [28,29]. In the present study, a small amount of stress magnitude does not indicate any significant effect compared to both restorative materials; however, an evaluation of their long-term behavior is still required [30]. 

This study also simulated conditions with flexible base materials at the bottom of the cavity. The clinician often opts to keep it as part of the restorative treatment when cavities are large and deep. When the flowable composite was simulated, higher stress levels compared to glass ionomer cement at the enamel tissue were reported, as reported in Table 2. At dentin margins, when the bulk-fill resin composite was layered up, the peaks were similar, and when Alk was layered up, the model showed smaller stress peaks.Bulk restorations have stimulated interest in clinicians due to its easier material placement and reduced clinical steps compared to conventional resin composites. Reduced volumetric shrinkage and lower polymerization shrinkage stress are described in literature. The insertion of newer polymerization modulators and monomers can relieve stress when incorporated inside bulk polymeric materials. In addition, their translucency and photo initiators allow their use in increments higher than 2 mm [33]. However, there are clinical situations when the tooth already presents flowable composite or glass ionomer cement inside the cavity [34]. Thus, the bulk material will behave in contact with a substrate different from dental tissue. This study supports that the evaluated Alk can be clinically proposed since lower stress peaks were observed in the restoration [35,36]. However, other studies are still necessary to investigate the immediate and long-term bond strength between the Alk and different materials and to assess any corroboration with this study’s findings [37].

With the limitations of a 3D finite element analysis, this study did not consider all the factors present in the oral medium [38], such as pH and temperature variations, possible incorporated defects in the adhesive layer or restorative material, and different chewing loads. In addition, all the materials were considered adhesively bonded with ideal bond strength and misfit. The food bolus was represented by just one volumetric body and does not represent the entire possibilities of patient’s diet and loading application.

## 5. Conclusions

With the limitations of the present study, the adhesively bonded Alkasite restorations showed a promising mechanical behavior with reduced stress magnitude when used above different base materials or as a bulk restoration for Class I posterior restorations.

## Figures and Tables

**Table 1:** Mechanical properties considered in the present simulation.

Material/Structure	Elastic Modulus (GPa)	Poisson Ratio
Enamel [18]	84.1	0.33
Dentin [18]	18	0.30
Bulk-fill resin composite [22]	12.0	0.25
Flowable resin composite [22]	8.0	0.25
Glass ionomer cement [17]	10.8	0.30
Alkasite [19]	13.0	0.3

**Table 2:** Tensile stress peaks (MPa) in enamel, dentin, and restoration, according to the restorative material(s).

Model	Region	Stress (MPa)
Bulk restoration in bulk-fill resin composite	Enamel	11.12
	Dentin	4.15
	Restoration	5.93
Flowable resin composite below the bulk-fill resin composite restoration	Enamel	11.27
	Dentin	4.26
	Restoration	5.98
Glass ionomer cement below the bulk-fill resin composite restoration	Enamel	11.25
	Dentin	4.26
	Restoration	5.99
Bulk restoration in Alkasite	Enamel	12.04
	Dentin	4.49
	Restoration	4.73
Flowable resin composite below the Alkasite restoration	Enamel	12.41
	Dentin	4.50
	Restoration	4.76
Glass ionomer cement below the Alkasite restoration	Enamel	12.23
	Dentin	4.51
	Restoration	4.74

## Data Availability

The data presented in this study are available on request from the corresponding author.
